# Editorial Changes

**Published:** 1851-01

**Authors:** 


*Editorial Changes.*—Prof. J. P. Garvin has relinquished the editorial charge of the Southern Medical and Surgical Journal, and is succeeded by his colleague in the Georgia Medical College, Prof. L. A. Dugas. This periodical has always been conducted with signal ability, and its character will not be likely to snffer in the hands of its present editor.

